# Accurate Forecasting of Emergency Department Arrivals With Internet Search Index and Machine Learning Models: Model Development and Performance Evaluation

**DOI:** 10.2196/34504

**Published:** 2022-07-20

**Authors:** Bi Fan, Jiaxuan Peng, Hainan Guo, Haobin Gu, Kangkang Xu, Tingting Wu

**Affiliations:** 1 College of Management Institute of Business Analysis and Supply Chain Management Shenzhen University Shenzhen China; 2 Faculty of Science University of St Andrews St Andrews United Kingdom; 3 School of Management Science and Engineering Dongbei University of Finance and Economics Dalian China; 4 School of Electromechanical Engineering Guangdong University of Technology Guangzhou China

**Keywords:** emergency department, internet search index, machine learning, nonlinear model, patient arrival forecasting

## Abstract

**Background:**

Emergency department (ED) overcrowding is a concerning global health care issue, which is mainly caused by the uncertainty of patient arrivals, especially during the pandemic. Accurate forecasting of patient arrivals can allow health resource allocation in advance to reduce overcrowding. Currently, traditional data, such as historical patient visits, weather, holiday, and calendar, are primarily used to create forecasting models. However, data from an internet search engine (eg, Google) is less studied, although they can provide pivotal real-time surveillance information. The internet data can be employed to improve forecasting performance and provide early warning, especially during the epidemic. Moreover, possible nonlinearities between patient arrivals and these variables are often ignored.

**Objective:**

This study aims to develop an intelligent forecasting system with machine learning models and internet search index to provide an accurate prediction of ED patient arrivals, to verify the effectiveness of the internet search index, and to explore whether nonlinear models can improve the forecasting accuracy.

**Methods:**

Data on ED patient arrivals were collected from July 12, 2009, to June 27, 2010, the period of the 2009 H1N1 pandemic. These included 139,910 ED visits in our collaborative hospital, which is one of the biggest public hospitals in Hong Kong. Traditional data were also collected during the same period. The internet search index was generated from 268 search queries on Google to comprehensively capture the information about potential patients. The relationship between the index and patient arrivals was verified by Pearson correlation coefficient, Johansen cointegration, and Granger causality. Linear and nonlinear models were then developed with the internet search index to predict patient arrivals. The accuracy and robustness were also examined.

**Results:**

All models could accurately predict patient arrivals. The causality test indicated internet search index as a strong predictor of ED patient arrivals. With the internet search index, the mean absolute percentage error (MAPE) and the root mean square error (RMSE) of the linear model reduced from 5.3% to 5.0% and from 24.44 to 23.18, respectively, whereas the MAPE and RMSE of the nonlinear model decreased even more, from 3.5% to 3% and from 16.72 to 14.55, respectively. Compared with each other, the experimental results revealed that the forecasting system with extreme learning machine, as well as the internet search index, had the best performance in both forecasting accuracy and robustness analysis.

**Conclusions:**

The proposed forecasting system can make accurate, real-time prediction of ED patient arrivals. Compared with the static traditional variables, the internet search index significantly improves forecasting as a reliable predictor monitoring continuous behavior trend and sudden changes during the epidemic (*P*=.002). The nonlinear model performs better than the linear counterparts by capturing the dynamic relationship between the index and patient arrivals. Thus, the system can facilitate staff planning and workflow monitoring.

## Introduction

### Background

The emergency department (ED), which provides instant and efficient medical services for patients all day, is one of the most important parts in the health care system [1]. Unfortunately, the ED as the main entrance in modern hospitals is now under the threat of overcrowding, which can lead to serious negative consequences, such as treatment delays, increased patient mortality, and financial losses [2]. The common causes of overcrowding are inadequate resource allocation and increased demand for ED services, particularly during the epidemic period [3].

The management of patient flow is a challenge faced by many EDs. The ability to accurately forecast the demand for medical service in EDs has considerable implications for hospitals, as it can improve staff and equipment resource allocation. Considering the high cost of purchasing new medical resources in a short time, it is more reasonable to develop an accurate forecasting model of ED patient arrivals, which could enable better matching of current resources and ED visits. By forecasting the level of demand for ED care in advance, medical staff have the opportunity to prepare for this demand, which can improve the ED service throughput, avoid overcrowding, and ensure the safety of patients [4].

### Prior Work

Previous studies mainly focused on the relationship between patient arrivals and the traditional variables, including the historical data of patient arrivals, calendar, weather, and holidays [4-12]. There have been many successful applications. However, the sudden and transient changes in people’s behavior cannot be captured by the traditional variables. This information should be applied to predict ED visits before such changes are noticed in the ED [4,11,13]. Recently, there has been an increasing interest to apply internet data to predict the behaviors and intentions of people in many areas, such as tourist arrivals, product sales, stock returns, and unemployment rate [14-16]. In health care, the weekly information report from Google Trends can be used for weekly influenza epidemic detection [17,18]. Moreover, internet data have been shown to be useful for predicting disease trends [17-19]. However, in some scenarios, the reliability of Google Trend is of concern as it is vulnerable to the mass media and statistical anomalies [20,21]. Dugas et al [22] studied the association between influenza rates and crowding metrics using the Google Flu Trends. However, only few studies have been published regarding the potential of internet data to improve ED visit forecasting. Ekström et al [4] monitored the visits to a special, regional medical website to predict the daily ED attendance with linear regression. Combining calendar, weather, and autoregressive (AR) terms, the least absolute shrinkage and selection operator (LASSO) regression was applied to forecast ED patient arrivals [11]. Ho et al [13] predicted ED patient volume in the Singapore General Hospital using multiple regression and publicly available Google data [13]. Moreover, they even developed a software suite to enable data visualization and prediction of patient arrivals, which is convenient for hospital management. Although these methods work well in their scenarios, there remains room for further improvement of the ED forecasting, neither limited to a specific region nor relying on expert experience to collect internet information. Moreover, the aforementioned studies are mainly based on linear model, and the possible nonlinearity may be ignored. In our paper, the nonlinearity is among patient arrivals and all the independent variables (eg, calendar, holiday, weather, and internet search index) are considered. A general method is, however, needed to overcome the aforesaid limitations.

### Objective

The objective of this study is to develop an intelligent forecasting system with a machine learning model and internet search index to provide accurate prediction of ED patient arrivals, to verify the effectiveness of the internet search index, and to explore whether nonlinear models can improve the forecasting accuracy. First, the internet search index was constructed from 266 search queries and verified as a novel variable by a systematic method. The data were generated from Google search queries, covering disease names, causes, symptoms, treatments, and others. The different types of information required by the patient, as reflected by the search query, might capture population-level interaction with events, such as infectious diseases, that traditional data sources alone may miss. The relationship between the internet search index and the ED visits was examined by Pearson correlation coefficient, Johansen cointegration, and Granger causality. Second, linear and nonlinear models were applied to predict ED patient arrivals with or without internet search index, respectively [4-12]. In addition, a nonlinear model, the extreme learning machine (ELM), was introduced because of its good generalization abilities and high prediction performance in flow prediction [23].

## Methods

### Overview

**Figure 1:**
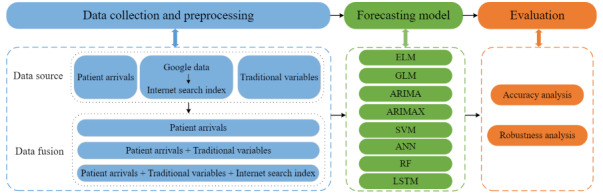
A framework of the intelligent forecasting system with the internet search index. ANN: artificial neural network; ARIMA: autoregressive integrated moving average model; ARIMAX: ARIMA with explanatory variables; ELM: extreme learning machine; GLM: generalized linear model; LSTM: long short-term memory; RF: random forest; SVM: support vector machine.

This study aimed to establish an intelligent system for predicting patient arrivals accurately and timely. The system consisted of 3 parts: data collection and processing, the establishment of forecasting model, and performance evaluation. In addition to the ED patient arrivals and traditional variables (weather, holidays, calendar), the internet search index, which extracted and integrated ED-related human behavior information scattered in Google search engines, was generated as a new variable. The correlation between the internet search index and patient arrivals was verified by Pearson correlation coefficient, Johansen cointegration, and Granger causality analysis. We then applied 8 forecasting models to predict ED patient arrivals, including ELM, generalized linear model (GLM), autoregressive integrated moving average model (ARIMA), ARIMA with explanatory variables (ARIMAX), support vector machine (SVM), artificial neural network (ANN), random forest (RF), and long short-term memory (LSTM) [24-33]. After that, their performances were evaluated in terms of accuracy and robustness analysis. The details of the intelligent system are shown in Figure 1.

### Data Collection and Processing

#### Data on ED Patient Arrivals

**Figure 2:**
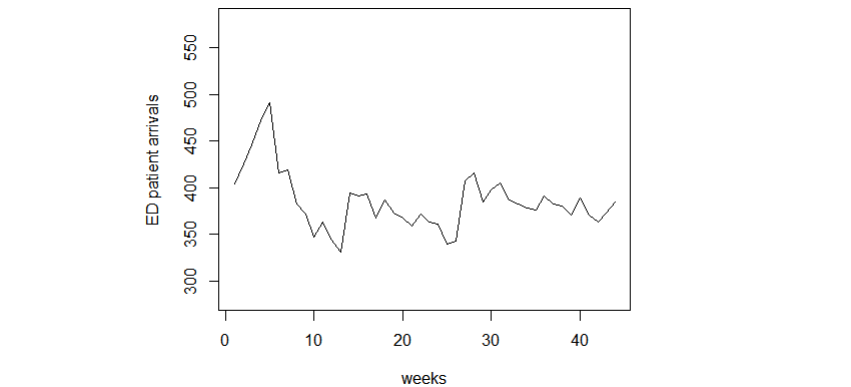
Weekly patient arrivals to ED. ED: emergency department.

In Hong Kong, patients can directly visit public ED without an appointment and be reimbursed for most medical expenses, so the ED is usually overcrowded. About 65% of patients walk-in and half of them are semiurgent or nonurgent [34]. The Cooperative Hospital is one of the largest public hospitals in Hong Kong, which is also the first one to receive patients with COVID-19. It has more than 4000 medical staff members and 1700 beds, and the hospital provides services for all residents living in Hong Kong, especially those in Kowloon. The number of ED patient arrivals from July 12, 2009, to June 27, 2010, had an annual flow of 139,910 ED visits, with an average of about 380 visits every day. The H1N1 pandemic broke out in Hong Kong during this period, which was a global epidemic before the outbreak of COVID-19. This ED provides 24/7 service for patients. As weekly scheduling arrangements have many applicable scenarios, we focused on the dynamic characteristics of weekly patient visits in this work. The hospital administrators use the ED weekly visits forecasting to optimize their human and material resources, as well as to enhance their preparedness for a crisis [10]. For the same purpose, some scholars forecasted ED weekly visits by considering the week of the year seasonality [3]. Every week is from Sunday to the next Saturday. For our analysis, all numerical data variables were converted to their corresponding weekly data by averages per week, which can represent the difference among weeks. In this way, the total number of holidays within a week was used to present the impact of this factor on patient arrivals. For temperature variables, we applied the data from the previous week to forecast patient arrivals in the current week. The data set has been examined and there were no heavy outliers. All variables were transformed with the minimum-maximum normalization technique before modeling. Therefore, the data of 51 weeks are the total data set. The data of the first 27 weeks were treated as the training data set and the rest as the testing data set. In the analysis, we divided the data set into 2 parts (60:40). We validated the model’s forecasting power with more testing data and the convenience of setting the split point at the beginning of the month. For example, if we were to predict ED patient arrivals for week t+1, then the data we applied included the number of ED patients at week t, the month of t+1 week, the highest and lowest temperatures at week t, and the total number of holidays for school and public holidays in the t+1 week. The search queries were from week t-6 to t from Google Trends. The normal and the outbreak conditions were considered in the training data. The weekly patient arrivals to the ED are shown in Figure 2.

#### Traditional Variables

**Figure 3:**
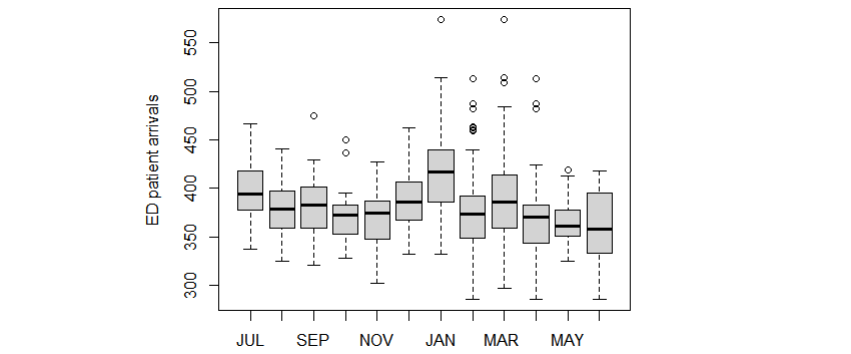
Boxplot of patient volumes of ED visits per month. ED: emergency department.

**Figure 4:**
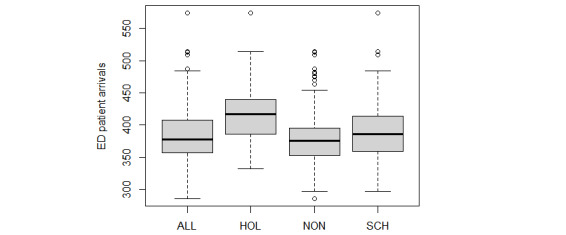
Boxplot of patient volumes in ED of holidays. ED: emergency department; HOL: holidays; NON: nonholidays; SCH: school closure.

According to previous studies [4-12], 5 exogenous variables were chosen as input and divided into 3 groups: calendar data (ie, months), weather data (ie, the daily highest and lowest temperatures), and holiday data (ie, school and public holidays). The daily highest and lowest temperatures were collected from Hong Kong Observatory. The public holidays included the following: The first day of January, the day preceding Lunar New Year’s Day, the first to third day of the Lunar New Year, Good Friday, the day following Good Friday, Easter Monday, the day following the Ching Ming Festival, Labour Day, the Buddha’s Birthday, the Tuen Ng Festival, Hong Kong Special Administrative Region Establishment Day, National Day, Chinese Mid-Autumn Festival, the Chung Yeung Festival, Christmas Day, and the first weekday after Christmas Day. The school summer holiday was from July 11, 2009, to August 31, 2009, and 2 school winter holidays were included: one from December 19, 2009, to January 3, 2010, and the other from February 10, 2010, to February 21, 2010. The boxplot of ED arrivals by month (Figure 3) shows that patient arrivals were much higher in July and January. Although the contribution of this variable is limited in our analysis, it was still considered because of its importance and the generalization of the model [4,7]. The boxplot of holidays shows that more patients visit the ED during public holidays (Figure 4).

#### Internet Search Index

##### Overview

In the Information Age, many patients prefer to search the internet to seek information about their problem before attending the ED. This was particularly the case during the flu outbreak in 2009-2010 [35]. Internet data can be monitored in near real time, showing the weekly dynamics of patient flow. Thus, sudden and transient changes in people’s behavior can be measured and used for prediction before such changes are actually noticed by the ED [4]. Internet data may also be a feasible surveillance tool for ED to prevent overcrowding. According to the data from Statcounter [36], the Google search engine has now become mainstream in Hong Kong, and thus using data from this search engine is conducive to the consistency of future forecasts. The search queries from Google Hong Kong were collected as internet data through Statistical Analysis Tools (Google LLC). In addition, the normalization of Google data only slightly affects the experiments because the data are renormalized in every iteration. However, because the normalization of Google Trends data is within a specific period, it is necessary to monitor the current values of the queries.

Google Trends is a common data-aggregating tool for measuring and analyzing Google search data, which can timely reflect the changes and trends in a society based on the popularity of specific Google search queries. The internet data collected had the same geographic area and period as ED patient arrivals. Data were collected by selecting “All categories” on Google Trends and Google Web Search between July 12, 2009, and June 27, 2010, in Hong Kong on the Google Trends official website. An internet search index was constructed by combining the ED-related search queries. The fusion method is a 4-stage process [12].

##### Step 1: Queries Generation

The selection of the initial queries is important to comprehensively collect internet information. As the current methods are mostly based on empirical intuition, the initial selection in this work was designed to expand the related search scope as much as possible. We defined and organized the information (Table 1) into 5 specific categories inspired by expert knowledge and well-studied papers: names of diseases, causes, symptoms, treatments, and others [13,22,37]. The initial queries were selected based on expert knowledge and information from the Hong Kong Department of Health. This includes, in particular, the experience of ED staff, the most common search queries in health-related references as well as the information on infectious diseases and virus surveillance from the Department of Health [13,22,37,38]. Results of some queries indicated that potential patients with specific conditions may visit the ED. For example, poor weather contributes to the development of numerous ailments, such as asthma. Claritin is a common antiallergic medication among patients with allergy. Massages can help with lumbar muscle strain, muscle atrophy, and migraine headaches. In Hong Kong, honey is one of the most popular health care remedies for curing sore throat. Ultimately, the initial 20 queries were selected. Hong Kong is a multicultural city, and both Chinese and English are often used in search engines. For a better understanding, the English translations of Chinese search queries are shown in parentheses.

**Table 1:** Initial search queries related to emergency department patients

Aspects	Index
Names	癌症 (*cancer*), 流感 (*influenza*), abortion, flu, h1n1 symptoms
Causes	天氣 (*weather*), 病毒 (*virus*), pregnancy, skin problem, tobacco
Symptoms	喉嚨痛 (*sore throat*), 發燒 (*fever*), 出汗 (*sweat*), infections
Treatments	克拉汀 (*claritin*), 按摩 (*massage*)
Others	蜂蜜 (*honey*), 醫生 (*doctor*), 冬季 (*winter*), depression

##### Step 2: Queries Expansion

A total of 20 basic queries were used as seed words. The related queries were recommended by Google Trends. These queries were then applied in the second-round search. This process was repeated until the queries became unavailable. A total of 268 search queries were collected by this process. As multiple comparisons are involved, the *P* value modified by false discovery rate was applied to make it hard to reject the null hypothesis.

##### Step 3: Queries Selection

The Pearson correlation coefficients were calculated between ED patient arrivals and the search queries. As the actual distributions of queries and patient arrivals are unknown, they were assumed to be normally distributed by convention [11]. Pearson correlation can help find some interpretable queries to ensure their information is useful for prediction. Considering that the actual visit is later than the online search behavior, it is necessary to test search queries with different lags. For every query, 7 Pearson correlation coefficients were generated from the data of 7 weeks before the forecast week, denoted as lag1 to lag7. Among them, we selected queries with the largest correlation coefficient no less than 0.30, which is calculated between ED patient arrivals and the search queries in the training data set. Finally, 9 queries were selected as shown in Table 2. Taking into account their lags, they were shifted (ie, previous queries moved to the corresponding rows of the current week) and summed to build the index.

**Table 2:** Maximum correlation coefficient of search queries from Google Trends.

Number	Index	Aspects	Lag^a^	Correlation coefficient	*P* value^b^
1	ginger	Treatment	1	–0.33	.02
2	swine flu^c^ symptoms	Disease	1	0.50	<.001
3	Infect	Symptom	1	0.36	.01
4	衛生署 (Department of Health)	Others	1	0.43	.00
5	fever	Symptom	2	0.31	.04
6	豬流感 診所 (swine flu clinic)	Disease	2	0.49	<.001
7	牙醫 (dentist)	Others	2	–0.32	.04
8	肠病毒 (enterovirus)	Disease	6	0.38	<.001
9	cough	Symptom	7	–0.42	<.001

^a^The unit of lag is week(s).

^b^The *P* value is modified by false discovery rate (significance level=.05).

^c^Swine flu is the nickname of H1N1 influenza in Hong Kong.

##### Step 4: Internet Search Index Construction

**Figure 5:**
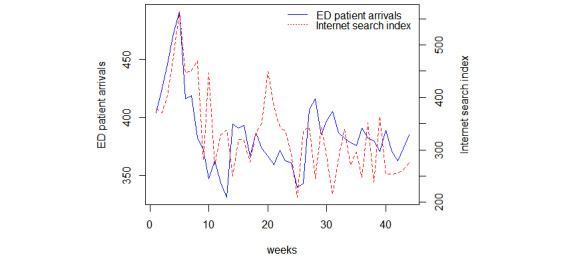
Trend of ED patient arrivals and internet search index. ED: emergency department.

To illustrate the contribution of the overall related internet information, the internet search index was employed by shifting and summing. In addition, the internet search index can effectively reduce the dimension of the data compared with the queries. According to the lag term, the 9 queries selected above were shifted in such a way that the previous queries moved to the corresponding rows of the current week. That is the reason why we applied search queries from at least one week before. All of the shifted search queries were summed to form the internet search index as a new time series. Although the fluctuation of the internet search index is greater than ED patient arrivals, it presents a similar trend to ED patient arrivals (Figure 5).

According to the aforementioned steps, 9 valuable queries were selected from a total of 268 queries, of which 4 were Chinese queries: 衛生署 (Department of Health), 豬流感 診所 (swine flu clinic), 牙醫 (dentist), and 肠病毒 (enterovirus). The remaining 5 were English queries (ginger, swine flu symptoms, infect, fever, and cough).

It has been observed that pandemic outbreaks can be captured by query terms [11,39]. The 3 queries here, swine flu symptoms, 豬流感 診所 (swine flu clinic), 肠病毒 (enterovirus), can be associated with the swine flu outbreak in 2009 and enteroviruses outbreak in 2010 in Hong Kong. In addition, the query 衛生署 (Department of Health) is related to ED visits. The Department of Health is the official hospital management agency in Hong Kong [38]. Its website provides reliable and comprehensive medical-related information and regularly issues outbreak alerts. The remaining queries (cold, infect, fever) belong to the common emergency services. Moreover, in Hong Kong, ginger is an effective medicinal spice used in daily life and widely used for the prevention and early treatment of cold. Therefore, “ginger” as a search query might suggest that the user is a potential ED patient. These queries capture local population–level health information and were translated into an internet search index. It provides information rarely found in traditional data sources, and thus, the forecasting of ED visits can be improved.

#### Extreme Learning Machine

For the forecasting model, we employed 8 different methods: ELM, GLM, ARIMA, ARIMAX, SVM, ANN, RF, and LSTM. Besides ELM, the others are well known. To the best of our knowledge, this is the first time that ELM has been applied for the prediction of ED visits.

ELM is a single hidden layer neural network algorithm [40-43]. It has been widely used in many fields because of simple mathematical description, lower computational burden, and faster learning speed [40]. The main feature of the ELM is that the algorithm can randomly generate the input weights and node biases. The least-square method is used to determine the output weight by simple matrix computations. These made it computationally attractive.

We used the sample data set 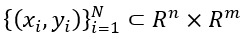, where *x_i_* is the input and *y_i_* is the output; *n* and *m* are the dimension numbers of input and output, respectively; and *N* is the number of samples. The forecasting model can be established using the ELM algorithm with *L* hidden neurons as follows:



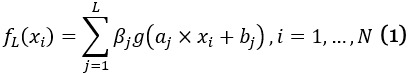



where *a_j_* and *b_j_* denote the input weight and the bias of the hidden layer, respectively; *g*(·) represents the activation function of hidden neurons; *βj* is the output weight representing the connected output neuron and the *j*th hidden neuron.

The following objective function is constructed to find the output weight *β*.



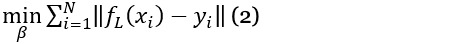





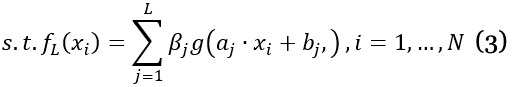



Equations (2) and (3) can be rewritten as:

H*β = Y* (4)

where *H* is the hidden layer output matrix.



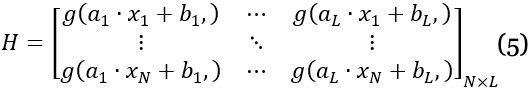





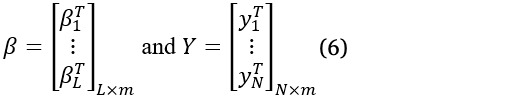



Through the least-squares method, the output weight *β* can be obtained as follows:



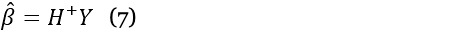



where *H*^+^ denotes the Moore-Penrose generalized inverse of matrix *H*. Using equations 1 and 7, the resulting ELM model can be estimated.

#### Evaluation Metrics

Two evaluation metrics were used to qualify the forecasting performance of different models: the root mean square error (RMSE) and mean absolute percentage error (MAPE). These are written as follows:



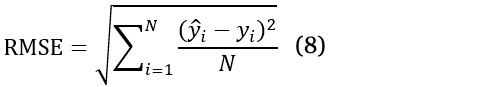





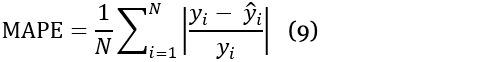



where *N* is the number of observations; *y_i_* is the real value; and 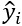 indicates the forecast value.

In addition, the Diebold-Mariano (DM) test was used to compare the forecast accuracy of forecast models [44]. The null hypothesis is that the reference model *re* is more accurate than the test model *te*. The DM statistic can be written as:



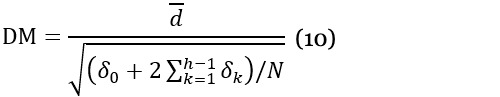



where 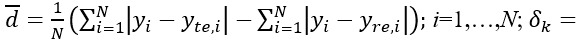



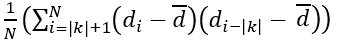
; *y_i_* is the actual value; and *y_re_*_,_*_i_* and *y_te_*_,_*_i_* are forecasting values of the reference and test models, respectively; *N* is the number of observations; *h*(>1) is *h*-step-ahead forecasts; 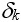 is the autocovariance of the loss differential at lag *k*. The loss differential time series *d_i_* is confirmed as stationary with the augmented Dickey-Fuller test.

## Results

### Relationship Between ED Visits and Internet Search Index

We first analyzed the association between the internet search index and ED patient arrivals with the Pearson correlation coefficient. We then also applied the Johansen cointegration and Granger causality to verify their relationship [45,46]. All 3 analyses were based on the training data set.

Initially, the Pearson correlation coefficients indicated that ED patient arrivals were significantly correlated with the internet search index (*r*=0.46, *P*=.002).

We next report the results of cointegration (Multimedia Appendix 1). Given the logarithmic form of the 2 variables to reduce the impact of outliers, a stability test was performed. These 2 time series were stably validated by the augmented Dickey-Fuller test [47]. The cointegration results indicated that ED arrivals and internet search index were cointegrated. The first hypothesis, r=0, tests for the presence of cointegration. As the test statistic exceeds the 1% level (66.22>23.52), we have strong evidence to reject the null hypothesis of *no cointegration.* The second test for r<1 against the alternative hypothesis of r>1 also provides evidence to reject r<1 because the test statistic again exceeds the 1% level (20.41>11.65). Thus, the cointegration results demonstrate that ED patient arrivals and the internet search index were cointegrated.

Meanwhile, the Granger causality test was applied to verify whether the internet search index is a predictor of ED arrivals. According to Multimedia Appendix 1, log(internet search index) is the Granger cause of log(patient arrivals). It indicates a causal relationship between internet search index and patient arrivals. We examined the relationship between internet search index and patient arrivals by Pearson correlation coefficient, Johansen cointegration, and Granger causality test. As the internet search index was correlated with ED patient arrivals, it can be included as a novel variable in the forecasting model.

### Forecasting Performance Evaluation

The forecasting models are linear and nonlinear models, including ELM, GLM, ARIMA, ARIMAX, SVM, ANN, RF, and LSTM. To test the predictive power of adding different data sources, the data can be classified into 3 types: “patient arrivals,” “patient arrivals + traditional variables,” “patient arrivals + traditional variables + internet search index.”

The prediction accuracy of the models is evaluated by MAPE and RMSE. As shown in Table 3, the results are promising because the models perform with a fairly high level of accuracy overall. It is obvious that combining the internet search index provides a higher prediction accuracy than both “patient arrivals + traditional variables” and “patient arrivals.” As stated earlier, internet search data can improve prediction.

**Table 3:** Prediction performance of weekly emergency department patient arrivals.

Models		Training			Testing	
		MAPE^a^ (%)	RMSE^b^	MAPE (%)		RMSE
**Patient arrivals**						
	ARIMA^c^	3.6	17.02	5.2		24.28
	ANN^d^	3.5	19.19	3.6		19.20
	SVM^e^	2.2	18.52	4.1		18.59
	RF^f^	2.5	19.36	3.0		19.67
	LSTM^g^	2.9	16.93	4.2		20.79
	ELM^h^	2.8	16.52	3.2		16.99
**Patient arrivals + traditional variables**						
	GLM^i^	3.2	16.79	5.3		24.44
	ARIMAX^j^	3.5	17.85	5.1		23.16
	ANN	3.4	16.10	4.0		18.48
	SVM	2.8	16.24	3.9		17.45
	RF	2.9	17.05	3.7		18.36
	LSTM	3.2	16.43	4.4		19.94
	ELM	2.7	13.17	3.5		16.72
**Patient arrivals + traditional variables + internet search index**						
	GLM	3.2	16.24	5.0		23.18
	ARIMAX	3.4	17.84	5.1		22.00
	ANN	3.0	14.51	3.3		15.45
	SVM	2.6	14.84	3.1		15.09
	RF	2.9	15.92	3.3		16.32
	LSTM	3.0	15.15	3.4		16.69
	ELM	2.6	13.10	3.0		14.55

^a^MAPE: average mean absolute percentage error.

^b^RMSE: root mean square error.

^c^ARIMA: autoregressive integrated moving average model.

^d^ANN: artificial neural network.

^e^SVM: support vector machine.

^f^RF: random forest.

^g^LSTM: long short-term memory.

^h^ELM: extreme learning machine.

^i^GLM: generalized linear model.

^j^ARIMAX: ARIMA with explanatory variables.

In particular, the performance of the models varies based on the value of the hyperparameters. The process of tuning the hyperparameters is performed to balance the relationship between optimal solution and regularization in the training data set, and thus to achieve the best generalization ability in the testing data set. As 2 commonly used parameter selection methods, trial-and-error and grid search guarantee good performance. Using these methods, we applied different models in this study. Both methods utilized different combinations of parameters and then built the best performance model with the selected parameters. With the trial-and-error method, the final selected GLM was fitted with Gaussian distribution rather than with other error distributions. The performance of ARIMA(X) is determined by the AR order (p), the degree of difference (d), and the moving average (MA) order (q). Autocorrelation function and partial autocorrelation were used to identify the value of AR and MA after verifying stationary by differencing the time series. The sigmoid activation function is applied in ANN. The specific values of hidden layer and hidden neuron are chosen from grid search. Similarly, the radial is used in SVM. A grid search is employed to select the number of costs, gamma, and epsilon. As for RF, the number of trees grown and the number of variables sampled at each split are decided through a grid search. Moreover, it is applied to tune the batch size, hidden units, and epochs. For ELM, the number of hidden nodes is set to 20, 120, and 150 for the “patient arrivals,” “patient arrivals + traditional variables,” and “patient arrivals + traditional variables + internet search index” data set, respectively. The kernel function is set to “satlins” for all data sets. Furthermore, as the initial weights were generated randomly, the parameter was decided by the average performance of the experiments (n<10) to ensure reliability. The optimal forms of ARIMA and ARIMAX were estimated by minimizing Akaike information criteria and Bayesian information criterion.

The ED experts informed that they had to increase additional medical staff members when configuration was mismatched by more than 18% [11]. Therefore, the aforesaid results indicate that there are 7 and 5 mismatch days for GLM without and with internet search index, respectively. ELM with internet search index had 2 mismatching days, which is the least value among all the forecasting models. Compared with “patient arrivals + traditional variables,” it can prevent 1 mismatching day theoretically.

Moreover, the best performance is achieved by ELM with independent variables of “patient arrivals + traditional variables + internet search index” in the training and testing data sets. It achieved an MAPE of 3%, with RMSE of 14.55. SVM also performed well, followed by ANN, RF, LSTM, and ARIMAX; GLM ranked last. The dynamic characteristic of the patient arrivals can be well represented by the ELM model.

The DM test was used to compare accuracy of forecasting models from a statistical point of view. The DM statistic results are shown in Table 4. With internet search index, when the ELM is applied as a test model with medium significance (*P*<.001), the model was superior to other forecasting models. By contrast, GLM had the lowest prediction performance among the 7 forecasting models. In addition, the performance of ELM, ANN, RF, SVM, and LSTM was better than that of ARIMAX and GLM. Therefore, nonlinear models may be more suitable for predicting the arrival of ED patients with the internet search index.

We next measured the DM test results between the reference model without internet data and the test model with internet data (Table 5). One of the critical findings is that the same models with internet data are better than those without internet data. Among all the models, neither GLM nor ARIMAX had a good performance, even with the internet data. All nonlinear models with internet data had higher accuracy than those without.

We assessed the robustness of the 7 forecasting models with or without the internet search index. All forecasting models were run 20 times using data set with different lengths. The robustness was evaluated by the SD of MAPE and RMSE. ELM was the most stable model with minimum SD of MAPE and RMSE (Table 6). By contrast, GLM was the most unstable forecasting model because it had maximum SD of MAPE and RMSE. The results also indicated that the forecasting models with the internet search index are more stable. Moreover, the robustness of nonlinear models is better than that of linear models. Compared with linear models, the rate of decline for nonlinear models is faster.

**Table 4:** DM^a^ test results of testing data set for same data set.

Test model			Reference model^b,c^										
			GLM^d^		ARIMAX^e^		ANN^f^		SVM^g^		RF^h^		LSTM^i^
**Patient arrivals + traditional variables**													
	ELM	2.8297 (<.001)		3.0624 (<.001)		2.012 (<.001)		1.8178 (<.001)		2.8481 (<.001)		2.1002 (<.001)	
	GLM			0.2935 (.31)		0.86595 (.11)		1.0707 (.06)		0.7643 (.09)		0.1663 (.54)	
	ARIMAX					0.64435 (.17)		1.0691 (.06)		0.3957 (.23)		0.4876 (.68)	
	ANN							0.13244 (.38)		0.2746 (.45)		1.9512 (.01)	
	SVM									0.5823 (.27)		0.8714 (.12)	
	RF											1.0045 (.08)	
**Patient arrivals + traditional variables + internet search index**													
	ELM	2.5062 (<.001)		3.79 (<.001)		2.0047 (<.001)		2.0325 (<.001)		2.0476 (<.001)		1.6659 (.02)	
	GLM			0.32675 (.30)		1.1462 (.12)		1.6064 (.07)		1.0467 (.09)		0.3647 (.64)	
	ARIMAX					1.7314 (.06)		2.2885 (.05)		1.5946 (.11)		1.2671 (.07)	
	ANN							0.14419 (.40)		0.2104 (.49)		1.2304 (.08)	
	SVM									0.2593 (.36)		1.4391 (.04)	
	RF											1.2992 (.06)	

^a^DM: Diebold-Mariano.

^b^The *P* value modified by false discovery rate is given in brackets. The significance level is .05.

^c^Values are presented as the Diebold-Mariano statistic (*P* value modified by false discovery rate).

^d^GLM: generalized linear model.

^e^ARIMAX: ARIMA with explanatory variables.

^f^ANN: artificial neural network.

^g^SVM: support vector machine.

^h^RF: random forest.

^i^LSTM: long short-term memory.

**Table 5:** DM test results of testing data set for different data sets.

Test model (with internet data)	Reference model (without internet data)^a^							
	GLM^b^	ARIMAX^c^	ANN^d^	SVM^e^	RF^f^	LSTM^g^	ELM^h^	
GLM	2.4848 (<.001)	0.4041 (.31)	0.2797 (.37)	0.2314 (.40)	1.8806 (.22)	2.1002 (.31)	0.8635 (.16)	
ARIMAX	1.5701 (.04)	2.5818 (<.001)	0.4968 (.28)	1.7698 (.12)	0.7756 (.20)	1.5748 (.24)	0.0337 (.51)	
ANN	2.2546 (<.001)	1.7547 (.02)	4.1945 (<.001)	2.8276 (<.001)	3.4393 (<.001)	1.2432 (.09)	4.4291 (<.001)	
SVM	2.244 (<.001)	1.7374 (.02)	6.0597 (<.001)	2.3394 (<.001)	1.6767 (.02)	0.8602 (.07)	3.2791 (<.001)	
RF	1.7886 (.02)	1.9591 (.02)	2.7599 (<.001)	1.8785 (.02)	2.3097 (<.001)	0.5800 (.05)	2.3075 (<.001)	
LSTM	0.8556 (.01)	3.3685 (<.001)	1.3620 (.07)	1.6441 (.04)	1.0560 (.12)	2.4263 (<.001)	1.9995 (.02)	
ELM	2.2546 (<.001)	1.7547 (.03)	4.1946 (<.001)	2.8276 (<.001)	3.4394 (<.001)	2.175 (.01)	4.4291 (<.001)	

^a^Values are presented as the Diebold-Mariano statistic. The *P* value modified by false discovery rate is in brackets. The significance level is .05.

^b^GLM: generalized linear model.

^c^ARIMAX: ARIMA with explanatory variables.

^d^ANN: artificial neural network.

^e^SVM: support vector machine.

^f^RF: random forest.

^g^LSTM: long short-term memory.

^h^ELM: extreme learning machine.

**Table 6:** Robustness analysis.

SD			Forecasting model												
			GLM^a^		ANN^b^		SVM^c^		ARIMAX^d^		LSTM^e^		RF^f^		ELM^g^
**Patient arrivals + traditional variables**															
	SD of MAPE^h^ (%)	2.5		1.0		1.0		1.7		1.7		1.2		1.0	
	SD of RMSE^i^	15.638		4.385		5.158		5.843		7.409		5.371		4.099	
**Patient arrivals + traditional variables + internet search index**															
	SD of MAPE (%)	2.4		0.8		0.7		0.9		1.3		0.8		0.7	
	SD of RMSE	15.212		3.577		4.008		5.681		5.797		3.985		3.370	

^a^GLM: generalized linear model.

^b^ANN: artificial neural network.

^c^SVM: support vector machine.

^d^ARIMAX: ARIMA with explanatory variables.

^e^LSTM: long short-term memory.

^f^RF: random forest.

^g^ELM: extreme learning machine.

^h^MAPE: average mean absolute percentage error.

^i^RMSE: root mean square error.

These analyses have revealed some interesting findings: (1) The forecasting performance is improved by the internet search index, which might reflect the behavioral trends of potential patients during the period of the H1N1 pandemic. (2) The accuracy of the ELM model was far superior than that of other forecasting models and the model captures the nonlinearities between the variables and ED patients. (3) Including internet search index results in more stable models and the proposed ELM was the most stable among the models.

## Discussion

### Principal Findings

As the number of patients increases continually, ED needs more information to make timely and target resource configuration strategies, thus preventing overcrowding and reducing social pressure. In the era of big data, internet data have been used in many areas and may help formulate new and appropriate measures to provide early warning signals to decision makers. In this study, we mainly focused on introducing internet data and nonlinear models to predict ED visits during the pandemic. The 3 contributions are summarized as follows. First, we compared the performance of linear and nonlinear models in the data set with or without internet search index to predict patient arrivals. The observed increase in forecasting accuracy could be attributed to internet data and kernel-based ELM. In addition, we investigated the performance metrics of previous studies. The visits to a special, regional medical website were monitored to predict the daily ED attendance with linear regression, with an MAPE of 4.8% [4]. Another linear model (LASSO) was employed, in combination with traditional variables, to reduce the MAPE and RMSE to 7.58% and 12.07 [11]. Recently, a multiple regression was applied with Google data to predict ED arrivals in the Singapore General Hospital. Its prediction curve indicated that MAPE was close to 8% [13]. Compared with the performance metrics, the minimum MAPE and RMSE obtained in our study were 3.0% and 14.55, respectively. The comparison reveals that our work is competitive. Although the compared studies have different scenarios, data, models, and environments, all found that the internet data can help in the prediction of ED patient arrivals. We further examined the accuracy and robustness from a statistics perspective. Second, a systematic method was applied to build the internet search index that reflects patient-related information as comprehensively as possible in search queries, including common diseases, possible causes, current symptoms, self-treatment, and others. Statistically, the effectiveness of the internet search index was also verified by Pearson correlation coefficient, Johansen cointegration, and Granger causality. Third, the characteristics of the ED visits during the outbreak of H1N1 pandemic were also modeled. The problem of ED overcrowding at such a serious time was more intense than at normal times, which could be a typical environment for the proposed method using the internet search index.

The proposed intelligent forecasting system predicts ED patient arrivals accurately and timely. The predictive power provided by the system stems from 2 parts. First, the internet search index that integrates with relevant internet search queries greatly contributes to the improvement of forecasting. According to the selected queries, the lag term of most queries was lag 1, indicating that patients are very likely to visit the ED within a week after identifying their symptoms. As we took internet data into account, if we extend the forecasting scale, it may miss some queries and fail to reflect the near–real-time trends and sudden changes. A trade-off between accuracy and time was thus necessary here. Ultimately, we made 1-week-ahead forecasting of ED patient arrivals. Whenever the new incoming data are higher than previously highest values, we first updated the internet data and then predicted the patient arrivals. More specifically, the new incoming higher value will be normalized as 1, meaning that we have obtained another extreme value and thus it is necessary to update the entire query data. The prediction model will also be retrained with the new query data. When the highest value remains the same after adding the new data, we simply add the new data to the existing ones. Second, kernel-based ELM can explore the nonlinearities in data to achieve better forecasting accuracy. It is a novel computational intelligence method based on single-hidden layer feedforward networks for regression and classification. The essence of ELM is that almost all nonlinear piecewise continuous functions can be used as the hidden-node output function, and thus, the feature mappings used in ELM can be very diversified to approximate arbitrary nonlinearity. Moreover, input weights and the hidden layer parameters are randomly generated independent of the training samples, and only the output weights are calculated through the least-square method. This characteristic leads to a significant improvement in the learning speed of ELM. Therefore, ELM can be applied in the identification of nonlinearity to forecast ED patient arrivals. The proposed system is utilized to forecast ED patient arrivals for a Hong Kong hospital. Our experimental results reveal that the forecasting system with ELM is significantly superior over the traditional linear models and some other nonlinear models. Meanwhile, the internet search index increases the forecasting power of all models. Therefore, this system will provide more information for the predicted values, and then well-matched resource allocation plans will be developed in real-time or near real-time per week.

### Limitations

The limitations of this paper are as follows. First, some patients may not be able to access the internet. As a result, their behaviors will not be recorded by the internet data. However, with the development of the mobile internet, it will be more convenient for people to obtain the information through search engines. Second, the queries we selected only contained Chinese and English. Thus, some internet searches in other languages are likely ignored; however, languages other than the aforesaid are less popular in Hong Kong. Third, the search queries we chose may be limited. Although we had 266 queries, some queries may have been missed. We believe that the search queries could be updated in another comprehensive query selection method over time. Moreover, the Granger causality may result in spurious causality. Finally, to ensure the reliability of the data from Google Trends, the influencing factors, including the mass media interference and the statistical anomalies, need to be considered. Other advanced methods in selecting informative queries, such as Spearman, will be seriously considered to improve the forecasting power of our method.

### Conclusions

This study supports the possibility of using internet data to predict ED visits during a pandemic and this is, to the best of our knowledge, the first study to use internet data and nonlinear models to predict ED visits. Compared with several related papers, we mainly focused on dynamic characteristics of patient arrivals during the H1N1 influenza, which was declared as a pandemic in Hong Kong in 2009 [4,11,13]. The problem of ED overcrowding at such that time was more serious than in normal times. Using the proposed framework, the ED-related human behavior information can be effectively extracted and introduced into the prediction model. In this study, an intelligent forecasting system was proposed with machine learning and internet search index to accurately predict weekly ED patient arrivals. Initially, we used a comprehensive and systematic method to build the internet search index with related search queries, which contained information about disease, causes, symptoms, and treatments. The relationship between the internet search index and ED patient arrivals was then verified by Pearson correlation coefficient, Johansen cointegration, and Granger causality. Finally, forecasting models were applied to different combinations of data with or without internet search index.

Our experimental results indicated that all of the forecasting models are more accurate when the internet search index is considered, as the internet data can timely reflect the changes and trends. Compared with other popular forecasting methods, the proposed kernel-based ELM model was more accurate and robust to present the nonlinearities between the variables and ED patients. In general, the performance of nonlinear models is better than linear models. This may imply that the dynamic relationship between variables and patient arrivals can be well represented by the nonlinear models. This intelligent forecasting system can be widely applied in other EDs, with the need to only update the internet search index according to regional or special requirements. It may also help ED managers to improve staff scheduling and allocate resources more effectively to prevent overcrowding by giving an early warning, especially during a pandemic like H1N1 or even during COVID-19 times.

Our future work will explore ED-related data from social media platforms, such as Twitter, Facebook, and Weibo, to investigate their impact on the ED patient arrivals. In addition, we plan to predict the ED patient flow with different severity levels. As there are 5 levels of ED patient arrival triages in Hong Kong, the relationship between these 5 levels of patient arrivals and internet information will be further studied. This will help ED managers develop a more flexible and targeted strategy to balance the need of different patients. Furthermore, the spatiotemporal changes in ED patient visits are worth studying in-depth. To further improve the accuracy of the forecasting model, deep learning algorithms will be of great interest in our future work, especially the ability to find efficient representations in large amounts of data.

## Appendix

Multimedia Appendix 1 Cointegration test and Granger causality test result.

## Abbreviations

ANN: artificial neural network
AR: autoregressive
ARIMA: autoregressive integrated moving average model
ARIMAX: ARIMA with explanatory variables
DM: Diebold-Mariano
ED: emergency department
ELM: extreme learning machine
GLM: generalized linear model
LASSO: the least absolute shrinkage and selection operator
LSTM: long short-term memory
MA: moving average
MAPE: average mean absolute percentage error
M-P: Moore-Penrose
RF: random forest
RMSE: root mean square error
SVM: support vector machine
