# Supplementary material for: Accurate Forecasting of Emergency Department Arrivals With Internet Search Index and Machine Learning Models: Model Development and Performance Evaluation
Source: JMIR Med Inform. 2022 Jul 20;10(7):e34504. doi: 10.2196/34504 (PMC9350824; doi:10.2196/34504)
Supplement: Multimedia Appendix 1 [file medinform_v10i7e34504_app1.docx]

| Augmented Dickey-Fuller test | | | | | | |
| --- | --- | --- | --- | --- | --- | --- |
|  | | *t* statistics | | | *P* value | |
| log(patient arrivals) | | –3.984 | | | .02 | |
| log(internet search index) | | –4.530 | | | .01 | |
| Cointegration between patient arrivals and internet search index | | | | | | |
| Rank | Eigenvalue | Trace statistic | Critical value(5%) | | | Critical value(1%) |
| r<= 1 | 0.39 | 20.41 | 8.18 | | | 11.65 |
| r = 0 | 0.67 | 66.22 | 17.95 | | | 23.52 |
| Granger causality test between patient arrivals and internet search index | | | | | | |
| Null hypothesis | | | | F-statistics | | *P* value |
| log(internet search index) does not Granger Cause log (patient arrivals) | | | | 5.14 | | .02 |
| log(patient arrivals) does not Granger Cause log(internet search index) | | | | 0.09 | | .77 |

Note: The *P* value is modified by false discovery rate. The significance level=.05
